# Direct Observation of the Biaxial Stress Effect on Efficiency Droop in GaN-based Light-emitting Diode under Electrical Injection

**DOI:** 10.1038/srep17227

**Published:** 2015-12-04

**Authors:** Jinjian Zheng, Shuiqing Li, Chilun Chou, Wei Lin, Feilin Xun, Fei Guo, Tongchang Zheng, Shuping Li, Junyong Kang

**Affiliations:** 1 Department of Physics, OSED, Fujian Provincial Key Laboratory of Semiconductor Materials and Applications, Xiamen University, Xiamen, 361005, China; 2 San’an Optoelectronics Co., Ltd., Xiamen, 361005, China

## Abstract

Light-emitting diode (LED) efficiency has attracted considerable interest because of the extended use of solid-state lighting. Owing to lack of direct measurement, identification of the reasons for efficiency droop has been restricted. A direct measurement technique is developed in this work for characterization of biaxial stress in GaN-based blue LEDs under electrical injection. The Raman shift of the GaN E_2_ mode evidently decreases by 4.4 cm^−1^ as the driving current on GaN-based LEDs increases to 700 mA. Biaxial compressive stress is released initially and biaxial tensile stress builds up as the current increases with respect to the value of stress-free GaN. First-principles calculations reveal that electron accumulation is responsible for the stress variation in In_*x*_Ga_*1−x*_N/GaN quantum wells, and then reduces the transition probability among quantum levels. This behavior is consistent with the measured current-dependent external quantum efficiency. The rule of biaxial stress-dependent efficiency is further validated by controlling the biaxial stress of GaN-based LEDs with different sapphire substrate thicknesses. This work provides a method for direct observation of the biaxial stress effect on efficiency droop in LEDs under electrical injection.

GaN-based light-emitting diodes (LEDs) are revolutionizing lighting applications toward realizing high efficiency. LED efficiency is increasingly becoming a major obstacle because of efficiency loss under high current injection with the advancement of solid-state lighting^1^,^2^. Numerous studies have been conducted to relieve efficiency droop via carrier density reduction^3^,^4^, electron confinement improvement, and hole injection enhancement^5^,^6^. Many mechanisms have been proposed to explain efficiency droop, such as Auger recombination^7^, carrier delocalization^8^, poor hole injection^9^,^10^, carrier leakage^11^, spontaneous emission reduction^12^,^13^, and non-radiative recombination^14^. Efficiency droop can be classified into two categories regardless of the absence of an agreed single cause of droop: (1) current-droop (J-droop), which is the decrease in efficiency as the driving current increases; and (2) temperature-droop (T-droop), which is the decrease in efficiency as temperature increases^15^,^16^.

Direct measurement of the influence of dominant parameters on efficiency droop in LEDs is rare because of extreme difficulties under electrical injection. In general, most physical quantities frequently depend on temperature and current. Indirect observation greatly restricts identification the causes of efficiency droop. Direct measurement of Auger electrons emitted from a semiconductor LED under electrical injection has been proposed recently to observe the signature of Auger electrons at high injected current densities higher than 50 A/cm^2^,^7^ which identifies the dominant efficiency droop mechanism^17^. However, Auger recombination only occurs at a high current injection (I∝*n*^>2^) and cannot account for efficiency droop at a low current range^4^.

Traditionally, carrier concentration *n* varies with temperature through Fermi–Dirac distributions under thermal equilibrium. When the junction temperature increases from 300 K to 600 K, only approximately 6% internal quantum efficiency (IQE) variation has been estimated through the APSYS modeling software based on the *ABC* model^4^,^18^. This weak relationship between the efficiency variation and thermal activation of the carrier concentration in the junction is irreconcilable with common experimental results, in which efficiency droop increases significantly as ambient temperature rises^15^,^16^. The issue on the effect of junction temperature on efficiency droop, except for thermal carrier activation, emerges based on experimental findings.

In dealing with semiconductors, the spontaneous influence of temperature on mechanical properties and electroconductivity should be considered. One of the most important mechanical properties is lattice constant, which is closely related to the semiconductor temperature. Given the distinct difference of thermal expansion coefficients between the well and the barrier of multiple quantum wells in the present LED active layers, a thermal mismatch stress variation is evident near the interface as temperature changes. Addressing the lattice constant variation involves revealing the piezoelectric effect in wurtzite III-nitride LEDs spontaneously. The effect means that lattice stress is changed because of electron injection. Both temperature- and current-dependent lattice constants would affect electronic structures, transition probability, and even quantum efficiency. Nevertheless, direct observation of the stress variation remains a challenge in LEDs under electrical injection.

This work aims to develop a direct measurement technique for stress in GaN-based blue LEDs under electrical injection. The stress variation, which involves compressive and tensile biaxial stresses, is characterized by a Raman shift under different currents. First-principles calculations are employed to discriminate the stress variation between temperature and current, and reveal the transition probability variations between interband quantum states and efficiency droop under different biaxial stresses. The stress variations are found to be attributed to the current injection based on the comparison of the simulated results with the stress variations and efficiency droop under different currents, and then reduction of the hole quantum states. As a result, efficiency droop occurs particularly at a lower current injection. Based on the stress-dependent efficiency variation rule, stress in LEDs is modified by controlling the thickness of mismatch sapphire substrates. Results of this study contribute in improving efficiency droop.

## Results

**Figure 1:**
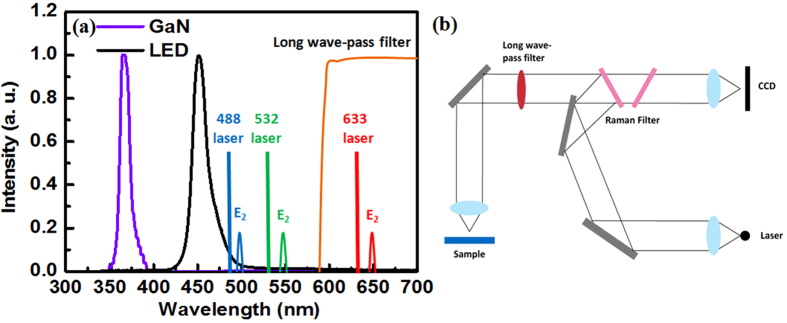
Schematic diagram. (**a**) Schematic diagram of the electroluminescence spectra from the GaN-based blue LEDs; the laser lines of 488, 532, and 633 nm; and the long wave pass filter with a pass wave of >600 nm. (**b**) Schematic diagram of the Raman microscope with an insertion of a long-wave pass filter.

Low scattered light intensity is found regardless if Raman scattering can be employed to identify stress in semiconductors. In particular, the power of the Raman scattered light is approximately 2–3 orders of magnitude less than that of electroluminescence from LEDs under current injection. The typical Raman microscope requires modification to avoid interference from electroluminescence of LEDs. Generally, electroluminescence from GaN-based blue LEDs consists of approximately 455 nm emission from multi-quantum wells and 500 nm to 600 nm defect-related yellow emissions. Raman scattering produces two possible outcomes: one appears in the lower energy side of the excitation laser line, which is called Stokes Raman scattering; and another is exhibited in the higher energy side, which is called anti-Stokes Raman scattering. In thermodynamic equilibrium, the Stokes Raman scattering peak is stronger than that of anti-Stokes scattering peak. Thus, Stokes Raman scattering is more commonly used in experimental measurement. Theoretically, a laser beam with a wavelength shorter or longer than the band edge emissions from semiconductors can be used for Stokes Raman scattering to avoid luminescence interference. For the GaN material, band edge emissions appear in the ultraviolet spectrum region and deep-ultraviolet laser is required. Given the uniqueness of ultraviolet optical devices, no commercial Raman scattering microscope is currently available. Visible laser lines, including the blue light of Ar^+^ 488 nm, the green light of 532 nm by frequency doubling 1,064 nm of Nd:YAG, and the red light of He–Ne 633 nm, are usually employed to detect Raman scattering. The E_2_ Stokes Raman phonon frequency shifts with biaxial stress in backscattering geometry with the laser beam incident on the (0001) surface of GaN-based blue LEDs^19^. An increase in the E_2_ phonon frequency with respect to unstrained GaN indicates compressive stress, whereas a decrease implies tensile stress. For stress-free GaN films, the E_2_ Raman phonon frequency is located at 567.1 cm^−1^ 20,21, which corresponds to wavelengths of 501.9, 548.6, and 656.6 nm for the laser lines of 488, 532, and 633 nm, respectively, as shown in Fig. 1(a). Although all of the three E_2_ modes are averted from the electroluminescence peaks of multi-quantum wells, the former two modes are at the edge of the electroluminescence peaks of multi-quantum wells or at the yellow band range. By contrast, the E_2_ mode for the 633 nm laser is far from the yellow band and can avoid electroluminescence interference even at a high current injection density, making this mode suitable for direct measurement. Furthermore, a long wave-pass filter with a pass wave greater than 600 nm is employed in the optical path of the Raman microscope to obtain an adequate signal-to-noise ratio, preventing electroluminescence from entering the spectrometer as the current injection. Therefore, a direct stress measurement technique under electrical injection has been designed by combining the Raman microscope with the 633 nm laser and a long wave pass filter.

**Figure 2:**
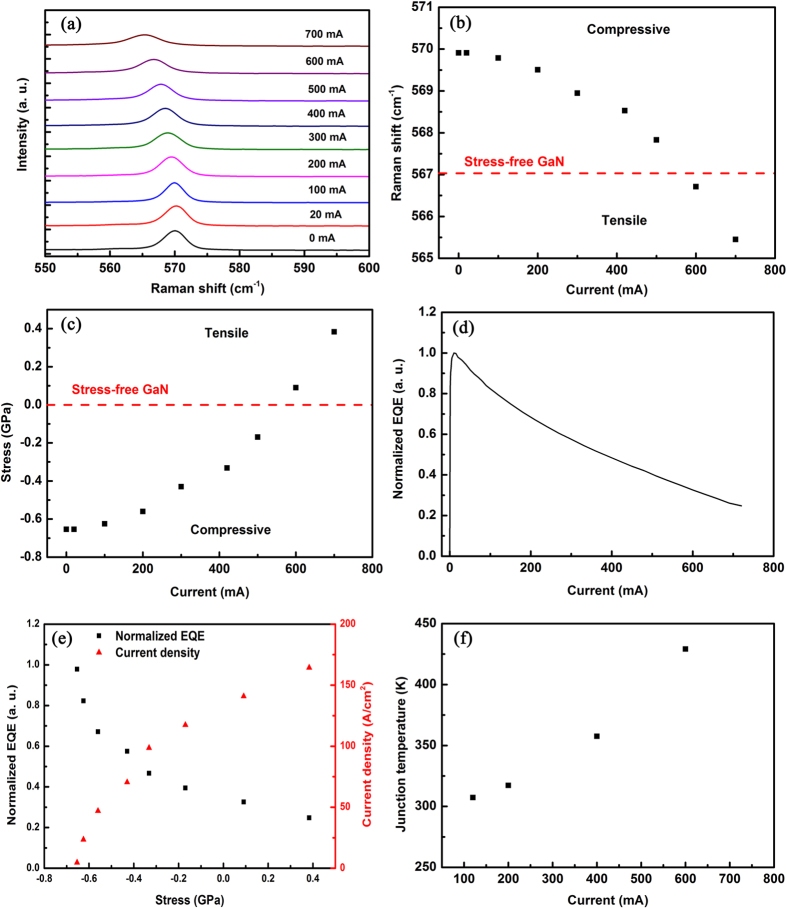
Raman spectra of the GaN-based LEDs. (**a**) Raman spectra of the GaN-based LEDs under currents ranging from 0 mA to 700 mA. (**b**) The Raman shift and (**c**) biaxial stress as a function of current. (**d**) Normalized external quantum efficiency (EQE) as a function of current. (**e**) Normalized EQE as a function of biaxial stress. (**f** ) Experimental junction temperature as a function of current.

The Raman spectra of GaN LEDs driving at different currents are designed to understand efficiency droop. Raman scattering experiment is performed using a Raman microscope (Renishaw UV-vis 1000) with a 633 nm laser when the samples of GaN-based LEDs are driving at currents from 20 mA to 700 mA at an ambient temperature of 300 K. The 100 cm^−1^ to 1,000 cm^−1^ spectra are collected through backscattering configuration with an incident light that is perpendicular to the sample surface. On account of the LED structure, the Raman shift is a comprehensive result originated from *n*-GaN layer, GaN barrier of QWs and *p*-GaN layer. Concerning the layer thickness, *n*-GaN with large thickness contributes in broadening of Raman Spectroscopy. Nevertheless, the active layer as a recombination center will induce large charge density causing most of voltage drops. It is believed that, among other layer containing GaN, GaN barrier in the active layer implicitly has a more sensitive Raman response accountable for the peak shift. The Raman spectra of GaN-based LEDs display the typical E_2_ mode at 569.9 cm^−1^ and the A_1_ mode at 736.2 cm^−1^ from the GaN film before current injection. As shown in Fig. 2(a,b), the E_2_ mode decreases as the current increases and reaches 565.5 cm^−1^ with a downshift of 4.4 cm^−1^ when the driving current on the GaN-based LEDs increases to 700 mA. Given that the E_2_ phonon frequency is affected by biaxial stress, the E_2_ phonon frequency shift Δ*ω* can be used to characterize the biaxial stress *σ*_*a*_ of the GaN layer according to the following relationship^20^:


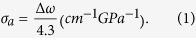


Based on the above relation, the biaxial stress under different current injections is determined through the E_2_ phonon frequency shift Δ*ω* with respect to unstrained GaN. Given that the E_2_ phonon frequency in the stress-free GaN is known to be located at 567.1 ± 0.1 cm^−1^ 20, the calculated compressive stress of 0.65 GPa in Fig. 2(c) is considered reasonable in GaN-based LEDs without current injection. Ensuring the release of biaxial compressive stress initially as the current increases and build up of biaxial tensile stress when the injection current is more than 500 mA is important, and the tensile stress finally increases to 0.38 GPa when the driving current increases to 700 mA. Stress is known to influence the material properties of III-nitrides, such as polarization field and electronic structures^22^,^23^. The directly measured distinct stress variation from compressive to tensile by approximately 1.03 GPa implies that more complications would accompany efficiency droop as the injected current increases from 20 mA to 700 mA.

The current-dependent external quantum efficiency (EQE) is compared with the stress variation to understand the origin of efficiency droop. The measured EQE as a function of the current is shown in Fig. 2(d). Similar to most blue LEDs^11^,^24^, approximately 70% normalized efficiency droop also occurs in the chip used in this study at 700 mA though a maximal EQE of approximately 65% at the injection current of 10 mA. With the current-induced relationship demonstrated in Fig. 2(c), the stress-dependent normalized EQE is derived and the results are plotted in Fig. 2(e). Notably, most EQE droops occur while the compressive stress of 0.65 GPa is released to stress-free. As the compressive stress becomes tensile, the EQE droop appears slower even under the biaxial tensile of 0.38 GPa. The EQE droop is known to be inevitable because not only biaxial stress changes but also temperature rises as the injection current increases. Accordingly, the p–n junction temperature is measured through the forward voltage change method to assess the effect of junction temperature on the EQE droop. As shown in Fig. 2(f), the junction temperature indeed increases with the current injection and reaches up to 450 K at a 700 mA current injection. The APSYS simulation shows that not more than 5% EQE variation emerges as the junction temperature increases by 150 K based on the SRH and Auger recombinations. Therefore, in conclusion, the primary EQE droop mechanism is the biaxial stress variation, especially at a low current range.

Given the 11% biaxial lattice mismatch and 32% thermal expansion coefficient between InN and GaN^25^, the biaxial stress is bound to change in the In_*x*_Ga_*1−x*_N/GaN MQW as the junction temperature increases. For the In_*x*_Ga_*1−x*_N quantum well with the In composition *x* of 0.18, the GaN barrier lattice suffers from tensile stress because of the smaller lattice constant of this barrier. According to the thermal expansion coefficient, the lattice contant *a* in GaN is thermally expanded from 3.189 to 3.194 Å. By linearly interpolating the thermal expansion coefficient from literature values for GaN and InN, the *a* value in In_0.18_Ga_0.82_N increases from 3.250 to 3.256 Å, which yeids reduction of lattice mismatch to 1.9%. It turns out that tensile stressis released (i. e. the compressive stress in GaN barrier increases) as the temperature increases. This temperature-dependent stress changing trend conflicts with the directly measured stress variation, indicating that the thermal-induced lattice mismatch cannot respond in the EQE droop.

**Figure 3:**
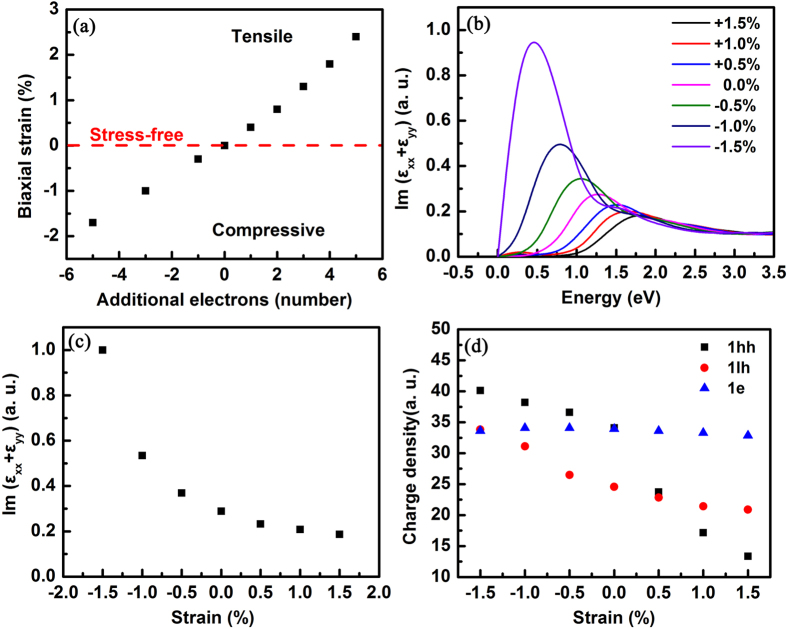
First-principles calculations. (**a**) Biaxial strain variation as a function of the additional number of electrons. (**b**) Calculated imaginary part of the dielectric function under different biaxial strains. (**c**) Intensity of Im (*ε*_xx_ + *ε*_yy_) as a function of biaxial strain. (**d**) Strain-dependent charge density for 1hh, 1lh, and 1e.

Traditionally, the piezoelectric effect is spontaneously addressed in wurtzite III-nitrides, implying that lattice stress would be changed because of electron accumulation. First-principles calculations are adopted to investigate lattice structures under different additional numbers of electrons to understand the lattice stress variation in the In_*x*_Ga_*1−x*_N/GaN MQW as electrons accumulate^26^,^27^. A geometric structure of (In_0.25_Ga_0.75_N)_2_/(GaN)_6_ MQW with a ratio of 1:3 between the well and the barrier is modeled to simplify the calculation and approach to the grown structures. The calculated lattice constant of the *a*-axis increases as the number of additional electrons rises, as well as in the *c*-axis. By contrast, the lattice constant of *a* decreases as the number of electrons reduces. Figure 3(a) demonstrates the biaxial strain variation as a function of the additional number of electrons. As the additional number of electrons decreases (i.e., the number of holes increases), biaxial compressive strain occurs in the MQW and extends to 1.7%, while the accumulated holes increase to 5. By contrast, biaxial strain becomes tensile in the MQW and extends to 2.3% as electrons accumulate, while the electrons accumulate to 5. The latter case normally occurs because the injected electrons should be larger than the injected holes because the net electron concentration from *n*-type GaN is more than one order of magnitude higher than that of the hole from the *p*-type in the GaN-based blue LEDs in this study.

Given that the samples used in this study are grown on sapphire substrates, the GaN lattice suffered from a strong biaxial compressive stress regardless if a low temperature buffer layer is used to release the mismatch stress. At a lower current region, the additional electron-induced biaxial tensile stress is not large enough to compensate the residual compressive stress; thus, the MQW suffers from a compressive stress resultant. At a higher current injection, the current-induced tensile stress is stronger than the residual compressive stress and dominates the MQW. Therefore, the biaxial compressive strain becomes tensile in the MQW when the current injection increases. Accordingly, the residual strain in a region from −1.5% to + 1.5% is chosen to model the In_*x*_Ga_*1−x*_N/GaN MQW for the first-principles calculations.

The first-principles calculations of the imaginary part of the dielectric function, carrier distribution, and electronic structures are performed to understand the biaxial strain mechanism that determines the optical properties and efficiency droop. The transition probability in the biaxial strained In_*x*_Ga_*1−x*_N/GaN is investigated by calculating the imaginary part of the dielectric function *ε*_2_ for a polarization of light parallel to the *c*-axis. The imaginary part of the dielectric function for optical absorption is computed using Fermi’s golden rule for the optical transition rate^28^. Figure 3(b) shows the imaginary part of the dielectric function Im (*ε*_xx_ + *ε*_yy_) that is proportional to the interband optical absorptions for the unpolarized light incident in the < 0001 > direction. The peak energies of the 1*h*-1*e* transition increase as the biaxial strain changes from compressive to tensile, which well agrees with the experimental blue shift result under the current injection^29^,^30^,^31^. As shown in Fig. 3(c), the peak intensity of Im (*ε*_xx_ + *ε*_yy_) for the band edge transition decreases dramatically as the biaxial compressive strain is released, and then declines slowly as the biaxial tensile strain builds up, whose tendency is consistent with the evident stress-dependent efficiency droop shown in Fig. 2(e). The transition probability is generally proportional to the wavefunction overlap and transition matrix element among the related states. The partial charge density of the quantum levels is employed to visualize the associated square wavefunction distribution. Accordingly, the 1*e*, 1*lh*, and 1*hh* levels have become the focus, as shown in Fig. 3(d). As the strain varies from the compressive state to the tensile state, the charge densities of 1*hh* and 1*lh* both decrease severely, while the charge density of 1*e* remains substantially unchanged. Furthermore, the transition matrix elements between 1*e*–1*lh* and 1*e*–1*hh* are calculated, and the results apparently increase slowly as the biaxial compressive strain is released and the biaxial tensile strain builds. Accordingly, less hole quantum states is responsible for the decrease in Im (*ε*_xx_ + *ε*_yy_) and the efficiency droop.

**Figure 4:**
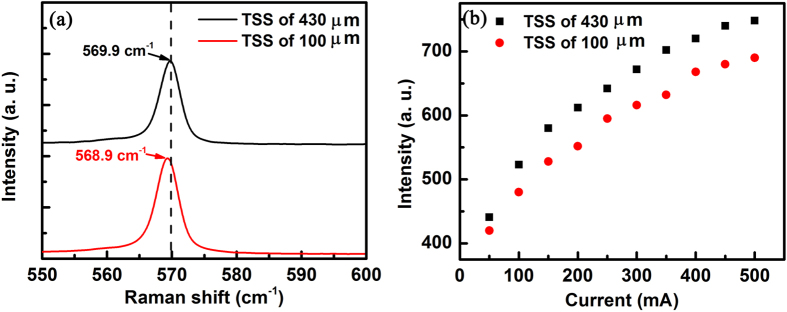
Controlling biaxial stress of GaN-based LEDs. (**a**) Raman spectra and (**b**) absolute light-output powers of devices for GaN LED grown on sapphire substrate thickness of 100 and 430 μm at room temperature with the injection current ranging from 0 to 500 mA.

The biaxial stress-related efficiency droop mechanism is further validated by thinning the sapphire substrate for the biaxial compressive stress release. The Raman shift is also directly measured on the GaN-based LEDs with 100 and 430 μm thickness of sapphire substrate (TSS). As shown in Fig. 4(a), the Raman shift of the GaN E_2_ mode of the GaN-based LEDs with 100 μm TSS is located at 568.9 cm^−1^ with a downshift of 1 cm^−1^ compared with 569.9 cm^−1^ for the 430 μm TSS, indicating a reduction in the biaxial compressive stress of GaN-based LEDs by 0.23 GPa according to Equation (1). The absolute light-output powers of devices are measured using an integrating sphere with the injection current ranging from 0 to 500 mA. As shown in Fig. 4(b), the light-output power averagely decreases by ~8% for the thinner sapphire substrate, confirming that biaxial compressive stress accumulation can improve external quantum efficiency. Efficiency droop can be modified by controlling the biaxial stress of GaN-based LEDs based on the rule of biaxial stress-dependent efficiency.

## Conclusion

Extensive studies have focused on the quantum efficiency of LEDs. A direct measurement technique is developed in this study for the stress in GaN-based blue LEDs under electrical injection, which provides important insights into the fundamental processes that are necessary for improvement of quantum efficiency. Characterized by Raman spectroscopy measurements, the Raman shift of the GaN E_2_ mode decreases as the current increases and reaches 565.5 cm^−1^ with a downshift of 4.4 cm^−1^ when the driving current on GaN-based LEDs increases to 700 mA, indicating that the biaxial stress vary by approximately 1.03 GPa. The largest EQE droop occurs while the compressive stress of 0.65 GPa is released stress-free. As the compressive stress becomes tensile, the EQE declines slower even under the biaxial tensile of 0.38 GPa. The first-principles calculation results show that the lattice of the MQW expands and the biaxial tensile strain reaches 2.3%, while the electrons accumulate to 5 because of the high current injection. The intensity of Im (*ε*_xx_ + *ε*_yy_) decreases dramatically as the biaxial compressive strain is released, and then slowly declines as the biaxial tensile strain builds up, whose tendency is consistent with the experimentally observed stress-dependent efficiency droop. According to the charge analysis, as the strain varies from the compressive state to the tensile state, the charge densities of 1*hh* and 1*lh* both greatly reduce, while the charge density of 1*e* remains invariant, indicating that less hole quantum states is responsible for the decrease of Im (*ε*_xx_ + *ε*_yy_) and the efficiency droop. The rule of stress-dependent efficiency is further examined by comparing the light-output powers of LEDs on sapphire substrates with different thicknesses. Based on the efficiency dependence on stress, stress control may produce a new degree of freedom in the design of LEDs with improvement with regard to efficiency droop.

## Methods

### MOCVD growth

The samples of GaN-based LEDs were grown on *c*-plane (0001) sapphire substrates via MOVPE (VEECO K465I 54 × 2”). The samples consisted of a 20 nm-thick GaN buffer layer, a 4 μm-thick unintentionally doped GaN layer, a 2 μm-thick *n*-GaN layer with an Si dopant concentration of 1.2 × 10^19^ cm^−3^, eight pairs of In_*x*_Ga_*1−x*_N/GaN MQWs with the composition *x* of approximately 0.18, and a 200 nm-thick *p*-GaN layer with an Mg dopant concentration of 9.4 × 10^19^ cm^−3^.

### Raman measurement

A Raman scattering experiment was performed using a Raman microscope (Renishaw UV-vis 1000) with a 633 nm laser. The spectra from 100 cm^−1^ to 1,000 cm^−1^ were collected through backscattering configuration with an incident light perpendicular to the sample surface.

### APSYS simulations

To investigate temperature dependent IQE, the simulations were been performed using the software package APSYS for experimental samples. The *p*-type GaN in the simulated LED is doped with ~ 2 × 10^19^ cm^−3^ acceptors. A SRH recombination lifetime of 100 ns, a Auger recombination coefficient of 10^−31^ cm^6^/s in quantum wells and percentage of screening effect of 50% were chosen for the simulation.

### First-principles simulations

The periodic supercell was composed of 2 × 2 × 4 arrays of primitive cells. The volume and shape of the supercell, and the internal positions of the atoms were initially defined by the GaN parameters, which were close to the practical epitaxy growth systems and were allowed to be simultaneously relaxed to the equilibrium. The additional neutralizing background charge was applied in the VASP simulations. The exchange-correlation function was described within the GGA. The pseudopotentials were specified through the PAW method. The effect of semicore Ga 3*d* electrons on the valence states was considered. The total energy of the system was relaxed to a minimum with a convergence criterion of 0.1 meV. Given an 8 × 8 × 4 Gamma-centered Monkhorst-Pack grid of *k*-points that sampled the Brillouin zone, a 500 eV cutoff energy was used to expand the electronic wavefunctions in a plane wave basis^32^.

## Additional Information

**How to cite this article**: Zheng, J. *et al.* Direct Observation of the Biaxial Stress Effect on Efficiency Droop in GaN-based Light-emitting Diode under Electrical Injection. *Sci. Rep.* **5**, 17227; doi: 10.1038/srep17227 (2015).
